# Improving molecular diagnosis of fabry disease: functional validation of novel splicing variants in *GLA*

**DOI:** 10.1186/s13023-026-04512-1

**Published:** 2026-08-13

**Authors:** Jingwen Zhou, Aiping Mao, Jie Feng, Na Hao, Fengxia Yao, Zhengqing Qiu, Danhua Li, Juntao Liu

**Affiliations:** 1 National Clinical Research Center for Women’s Health and Obstetric & Gynecologic Diseases, Department of Obstetrics and Gynecology, Peking Union Medical College Hospital, Chinese Academy of Medical Sciences & Peking Union Medical College, Beijing, China; 2 Department of Research and Development, Berry Genomics Corporation, Beijing, China; 3 The Laboratory of Clinical Genetics, Medical Research Center, Peking Union Medical College Hospital, Chinese Academy of Medical Sciences & Peking Union Medical College, Beijing, China; 4 Department of Pediatrics, Peking Union Medical College Hospital, Chinese Academy of Medical Sciences & Peking Union Medical College, Beijing, China

**Keywords:** Fabry disease, Pathogenicity, Minigene assay, Aberrant splicing, Frameshift

## Abstract

**Background:**

Fabry disease (FD) is an X-linked lysosomal storage disorder caused by pathogenic variants in the *GLA* gene. Although genetic testing is the gold standard for FD diagnosis, the identification of numerous splicing variants of unknown pathogenicity poses a significant diagnostic challenge. This uncertainty limits the clinical utility of sequencing in guiding patient management and therapeutic intervention.

**Methods:**

A combination of in silico prediction tools and in vitro minigene splicing assays was utilized to explore the pathogenicity and underlying molecular mechanism of novel deep non-coding variants identified via long-read sequencing in clinically suspected but genetically unsolved patients.

**Results:**

Functional assays revealed that the large deep-intronic insertion c.640-613ins1715 fundamentally disrupts local splicing pattern and introduces new non-canonical splicing sites. It induces a complex array of aberrant events, including partial exon skipping, intron retention, and pseudoexon inclusion, generating five distinct abnormal transcripts alongside residual normal transcript. These aberrant transcripts introduce frameshifts predicted to yield truncated, non-functional proteins. Consequently, this variant was definitively classified as Pathogenic. Conversely, neither the two deep-intronic single nucleotide variants (c.640-363C > T and c.639+761 G > A) nor their cis configuration exhibited aberrant splicing in our minigene system. However, due to the proband’s later-onset FD phenotype, ambiguous population frequencies (gnomAD), and the potential for tissue-specific splicing missed by in vitro models, both variants remain classified as VUS.

**Conclusions:**

This study expands the mutational spectrum of the *GLA* gene and underscores the complexity of deep-intronic variants. Our findings highlight the critical role of robust functional splicing assays in interpreting variant pathogenicity, establishing accurate genotype-phenotype correlations, and ultimately facilitating precision medicine for FD.

**Supplementary Information:**

The online version contains supplementary material available at https://doi.org/10.1186/s13023-026-04512-1.

## Introduction

Lysosomal storage disorders (LSDs) are a group of inherited metabolic diseases characterized by lysosomal dysfunction due to the progressive accumulation of undegraded macromolecules [1]. With over 60 identified subtypes, most LSDs are autosomal recessive inherited diseases, though some are X-linked such as Fabry disease (FD, OMIM 301,500) [2]. FD is caused by pathogenic variants in the *GLA* gene, which spans 12 kb and is rich in Alu repeat elements [3, 4]. The *GLA* gene encodes the enzyme α-galactosidase A (α-Gal A). Deficiency of α-Gal A leads to the progressive accumulation of macromolecules, such as globotriaosylceramide (Gb3) and its deacylated derivative, lyso-Gb3. This accumulation can trigger irreversible damage in multiple organs, including the kidneys, heart, blood vessels, and peripheral nervous system [5]. In hemizygous males, FD is clinically classified into severe classic and late-onset types. Patients with the classic form have absent or severely reduced ɑ-Gal A activity (<1% of normal), which leads to prominent clinical symptoms in childhood, such as neuropathic pain, corneal verticillata, acroparesthesia, and angiokeratomas. If untreated, the disease can progress to hypertrophic cardiomyopathy, arrhythmias, strokes, and renal failure [6]. In contrast, patients with the later-onset form typically have residual α-Gal A activity and exhibit clinical manifestations primarily in a single organ, usually the heart, during adulthood [7].

Disease-specific treatments for FD, primarily enzyme replacement therapy and chaperone therapy, can prevent severe organ damage and fundamentally alter the natural history of the disease [8]. Consequently, a timely and accurate diagnosis is critical. In general, FD diagnosis is established by detecting extremely low or undetectable α-Gal A enzymatic activity, followed by confirmatory genetic testing. However, in some heterozygous females, enzyme activity levels can overlap with normal values. Therefore, molecular genetic testing remains the gold standard for definitive FD diagnosis, especially in females [5]. Currently, the genetic diagnosis of FD relies on multiple sequencing strategies, ranging from traditional Sanger sequencing to advanced, exome, genome, and long-read sequencing technologies. To date, more than 1000 *GLA* variants have been registered in the Human Gene Mutation Database (HGMD). Notably, approximately 5% of these are classified as putative splicing variants. Confirming the pathogenicity and unraveling the precise molecular mechanisms of these variants is essential for establishing definitive clinical diagnoses and guiding therapeutic interventions. Because in silico computational algorithms often exhibit limited reliability in predicting complex splicing alterations, especially for deep-intronic variants, in vitro functional minigene assays have emerged as an indispensable and powerful platform for accurately assessing their transcriptional impact.

Our laboratory previously conducted a retrospective clinical study, involving 48 clinically diagnosed FD patients [9]. We developed the comprehensive Analysis of FD (CAFD) assay, which combines long-range PCR with Pacific Biosciences (PacBio) long-read sequencing (LRS). The CAFD assay achieved a diagnostic yield of 97.92% (47/48), which was 2.1% higher than that of conventional Sanger sequencing. Notably, the CAFD assay identified a novel deep intronic pathogenic variant (c.640-613ins1715) and two deep intronic variants of uncertain significance (VUS) in a cis configuration (cis c.639+761 G > A; c.640-363C > T), according to the guidelines of the American College of Medical Genetics and Genomics (ACMG). However, the pathogenicity of these variants has not yet been confirmed by functional studies. The presence of VUSs poses a significant clinical barrier, limiting the ability of genetic testing to provide definitive diagnoses and accurately guide therapeutic management. [9]. In this study, a combination of in silico prediction tools and in vitro minigene splicing assays was applied to explore the underlying pathogenic molecular mechanisms of the splicing variants identified via long-read sequencing.

## Methods

### Patient cohort

A total of two clinical suspected FD patients enrolled in this study were derived from a retrospective cohort study of 48 unrelated patients suspected of FD, recruited at Peking Union Medical College Hospital from 2004 to 2023 [9]. Using the CAFD assay, proband 1 (F47-1) and proband 2 (F48-1) were identified to carry deep intronic variants of GLA gene (National Center for Biotechnology Information [NCBI] ref-seq: NM_000169.3), which were further selected for functional validation assays due to their potential pathogenicity (Table 2). The variant c.640-613ins1715 in proband 1 is absent from the gnomAD population database and ClinVar database. The c.640-363C > T and c.639+761 G > A in cis configuration were identified in proband 2. The frequency of variant c.640-363C > T in gnomAD is 0.0004071, and the frequency of variant c.639+761 G > A in gnomAD is 0.00008050. These two variants were all registered in ClinVar database due to our published literature [9]. Notably, pathogenicity of c.640-363C > T and c.639+761 G > A in cis configuration were unclear. Furthermore, two patients were missed by conventional genetic testing, including Sanger sequencing and multiplex ligation-dependent probe amplification (MLPA). The pathogenicity of variants was assessed in accordance with the guidelines of the ACMG [10].

### Variant analysis

SpliceAI (https://spliceailookup.broadinstitute.org/) was utilized to predict potential cryptic splice sites that could result in abnormal splicing events [11]. Additionally, the Rare Disease Data Center (RDDC) RNA Splicer (https://rddc.tsinghua-gd.org/) was used for online analysis of splice variants [12]. Both tools evaluate the strength of newly formed or cryptic splicing sites in *GLA* variants.

### Minigene splice assay

To assess the potential impact of the identified novel deep intronic variants, minigene splice assays were performed as previously described [13]. A total of six minigene constructs were generated. For the *GLA* gene variants, including c.640-613ins1715, c.639+761 G > A, c.640-363C > T and the double variants in cis configuration (c.639+761 G > A and c.640-363C > T), wild-type and mutant genomic DNA fragments were amplified, each spanning exon 4 (92bp)-intron4(1719bp)-exon5(162bp). These fragments were individually cloned into the pcDNA3.1 minigene vector.

Nested PCR primers were designed for each target fragment Table 2. Nested PCR was performed to generate the target genomic fragments. The fragments and corresponding vectors were then subjected to enzymatic digestion, purification, ligation to construct recombinant vectors. These recombinant vectors were transiently transfected into cultured Human embryonic kidney 293T and Henrietta Lacks (HeLa) cells for expression. Total RNA was isolated from the cultured cells, and then cDNA was synthesized. The region of interest was amplified from the cDNA, and the amplified products were subjected to Sanger sequencing to verify alternative splicing events.

## Results

### Study subjects

Two patients with clinical suspected FD were analyzed in this study (Table 1). Proband 1, a male presenting with the classic FD and extremely low ɑ-Gal A activity, was found via CAFD to harbor a novel, de novo, large insertion (c.640-613ins1715) within intron 4. As previously reported, this structural variant likely resulted from an AluSx-mediated genomic rearrangement [9]. Proband 2, a female presenting with a late-onset FD and residual ɑ-Gal A activity, was identified by CAFD as carrying two novel, maternally inherited deep-intronic variants in a cis configuration (cis c.639+761 G > A; c.640-363C > T). Given that all three identified variants were novel non-coding alterations, a total of four specific minigene constructs were designed and generated to functionally elucidate their impact on pre-mRNA splicing (Table 2).

**Table 1:** The baseline characteristics and molecular diagnosis results of 2 probands recruited in this study

Family member	Sample ID	Gender	Year of diagnosis	Age of diagnosis	Enzyme activity	CAFD	Origin
Proband 1	F47-1	Male	2016	12	0.7	c.640-613ins1715	De novo
Mother	F47-2	Female	NA	NA	NA	NV	NA
Sister	F47-3	Female	NA	NA	NA	NV	NA
Proband 2	F48-1	Female	2008	4	19.1	cis c.640-363C > T,c.639+761 G > A	Mat
Mother	F48-2	Female	NA	NA	NA	cis c.640-363C > T,c.639+761 G > A	NA

NA was used as an abbreviation for Not applicable; NV was used as an abbreviation for no variants; Data for baseline characteristics were derived from our previously published study [9]; The reference range was 29.0–64.4 nmol/ml for α-gal A activity

**Table 2:** Four minigene constructs generated for deep intronic variants

No.	Gene	Variants	Intron	Minigene assay			SpliceAI prediction	
				Splicing outcome	RNA effect	Protein effect	Δ score (acceptor gain)	Δ score (donor gain)
1	*GLA*	c.640-613ins1715	Intron4	a: Normal transcript	No effect	No effect	NA	NA
				b: Retention of intron4 (304bp+99bp)	r.639_640ins403	p.Lys213_Pro214insValArg*		
				c: Retention of intron4 (15bp+351bp)	r.639_640ins366	p.Lys213_Pro214insValArg*		
				d: Retention of intron4 (284bp) and partial deletion of exon5 (7bp)	r.639_646del7ins366	p.Lys213_Pro214insValArg*		
				e: Retention of intron4 (262bp) and partial deletion of exon5 (97bp)	r.639_736del97ins262	p.Lys213_Pro214insValArg*		
				f: Retention of intron4 (90bp) and partial deletion of exon5 (20bp)	r.639_659del20ins90	p.Lys213_Pro214insValArg*		
2	*GLA*	cis c.640-363C > T, c.639+761 G > A	Intron4	Normal transcript	No effect	No effect	NA	NA
3	*GLA*	c.640-363C > T	Intron4	Normal transcript	No effect	No effect	0 (−376bp)	0
4	*GLA*	c.639+761 G > A	Intron4	Normal transcript	No effect	No effect	0.01 (−105bp)	0.01(−161bp)

NA was used as an abbreviation for Not Applicable

### Splicing effect of the deep intronic large insertion

**Fig. 1:**
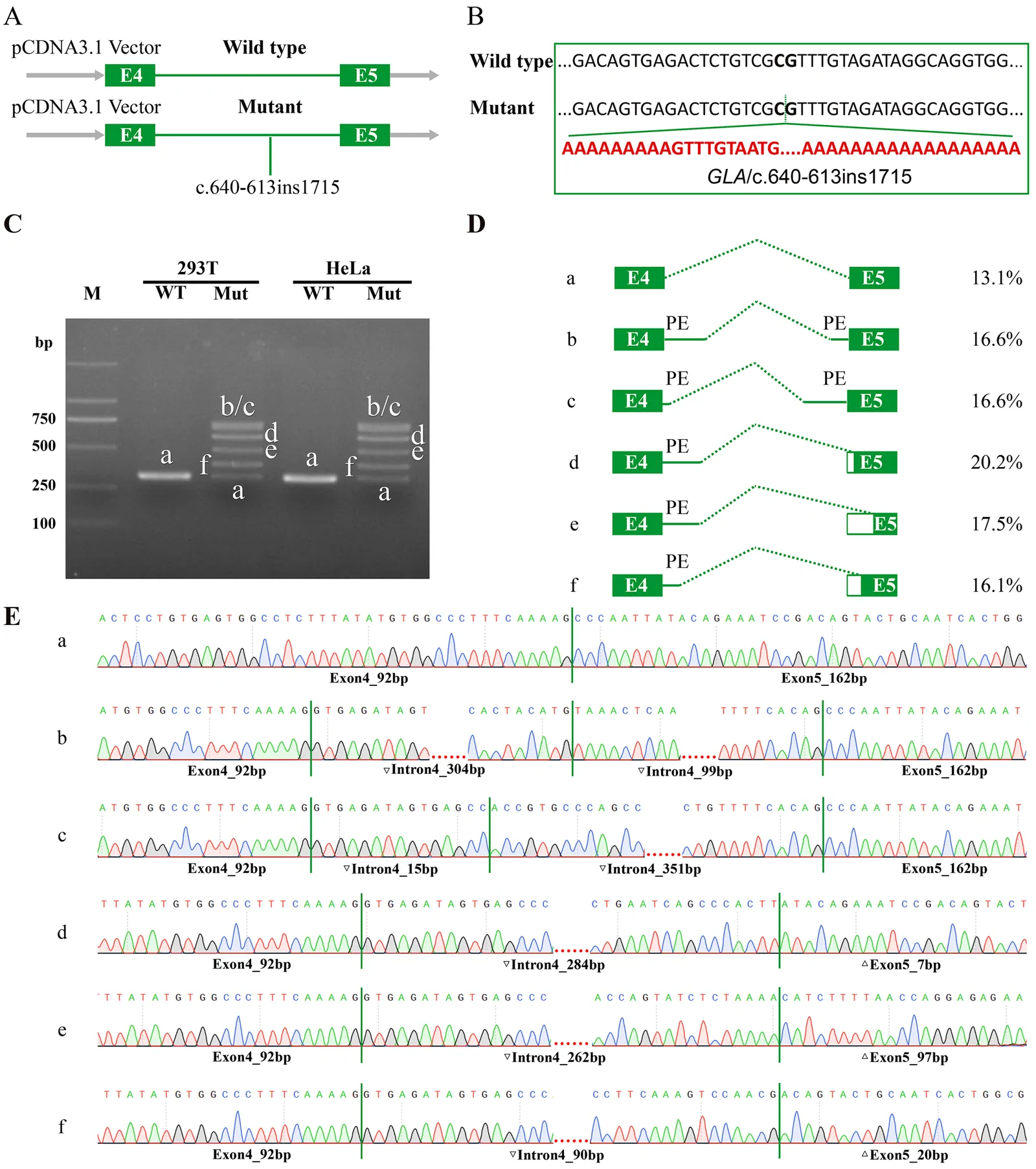
Minigene assay and splice defects caused by c.640-613ins1715in intron 4. (**A**) The schematic diagram of minigene constructs of wild type and the mutant. The green color shows fragments of wild type and the mutant genomic DNA and the gray color shows the pCDNA3.1 vector. (**B**) Genomic location of AluSx-mediated insertion. (**C**) RT-PCR products from 293 T and hela cells analyzed by agarose gel electrophoresis. (**D**) Schematic representation of splicing defects of variants. The solid lines represent pseudoexons (PEs). (**E**) Sanger sequencing chromatograms of RT-PCR products.∇, PEs; Δ, exon skipping

The variant c.640-613ins1715, located within the deep intronic AluSx element of the *GLA* gene, was first identified in proband 1 in our previous literature [3, 9, 14]. This variant is absent from the gnomAD population database, indicating its rarity. This novel, de novo variant was classified as pathogenic according to ACMG guidelines. However, its specific impact on splicing remained unclear. To investigate this, the minigene splicing assay in vitro was performed (Table 1). Genomic fragments of the wild-type and mutant alleles, spanning from exon4 (92bp) and downstream (162bp), were cloned into the pCDNA3.1 vector (Fig. 1A and B). Primers were listed in Table 2. The percentage of spliced products was quantified based on agarose gel electrophoresis. For the wild-type minigene, a single RT-PCR product representing the correctly spliced transcript was visualized by agarose gel electrophoresis (Fig. 1C), and confirmed by Sanger-sequencing (Fig. 1D). In contrast, the mutant minigene yielded six distinct transcript isoforms including five aberrant and one normal (Fig. 1C).

**Fig. 2:**
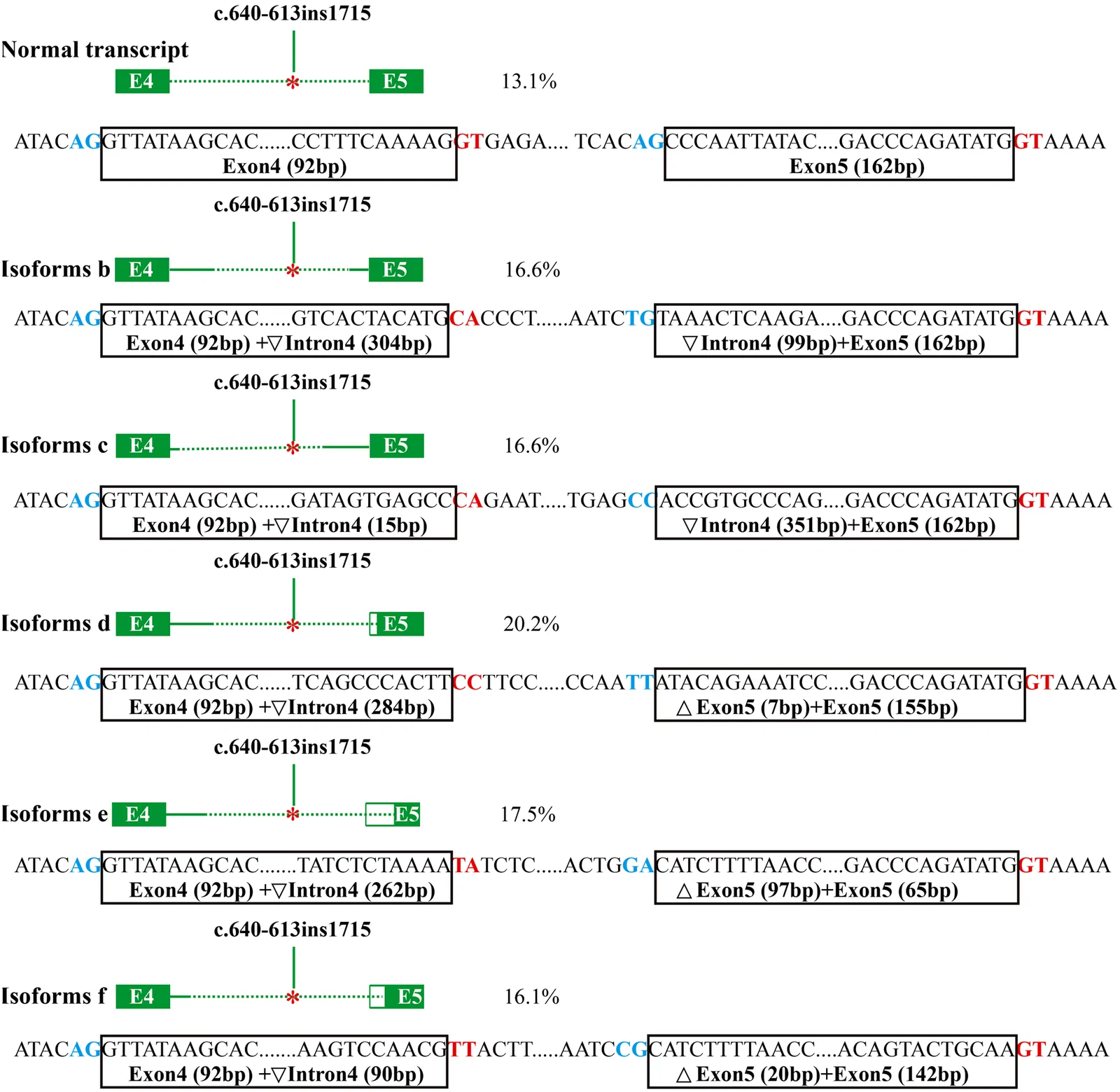
Canonical GT/AG splice sites in the normal transcript and the activation of cryptic splice sites in aberrant transcripts. The red and blue text indicate the donor and acceptor splice sites, respectively

Sanger sequencing confirmed that isoforms b and c retained a partial sequence of intron 4 between the canonical exon 4 and exon 5 junctions, generating pseudoexons (PEs) (Fig. 1D). Isoforms d, e, and f exhibited not only partial intron 4 retention but also partial skipping of exon 5 (Fig. 1D). Based on transcript quantification, the five aberrant transcripts accounted for 86.9% of the total transcription. The correctly spliced transcript accounted for the lowest proportion, at only 13.1%. The predominant transcript was isoform d, accounting for 20.2% of total expression, which was characterized by a 284bp intron4 retention and a 7bp exon5 deletion. The normal transcript utilized the canonical splicing sites GT/AG splice sites, the aberrant transcripts resulted in the activation of cryptic splicing sites (Fig. 2). These splicing defects caused frameshifts, and all five aberrant transcripts are predicted to generate a truncated protein, p.Lys213_Pro214insValArg* (Table 2). These results correlated well with the classic phenotype observed in proband 1, who was a 12-year-old male with no family history of FD and low ɑ-Gal A enzymes activities (0.7 nmol/mL). He clinically presented with acroparesthesias and refractory neuropathic pain, accompanied by skin blanching and decreased skin temperature, with a gradually increasing frequency of episodes.

This variant c.640-613ins1715 was located in the deep intronic AluSx element region of the *GLA* gene [9]. Notably, the *GLA* gene is particularly rich in Alu repeat elements [4]. Alu-mediated large rearrangements at least 0.2 kb in the intron4 of *GLA* gene are summarized in Figure S1 and Table S2. To the best of our knowledge, this is the first pathogenic case of a large insertion (≥200 bp) in the deep intronic region of *GLA* intron4 that is mediated by an Alu element. Furthermore, a total of 20 pathogenic and likely pathogenic (P/LP) variants within intron4 have been registered in the Fabry database (http://fabry-database.org/) and ClinVar Database (Table 3 and Figure S2). Among these, 50% (10/20) involve canonical splice sites-specifically the conserved GT donor sites at the 5’-end and AG acceptor sites at the 3’-end and are predicted to cause aberrant splicing without functional studies because they impact highly conserved sequences determining exon-intron boundaries [33, 34]. The remaining 10 variants (50%) were located outside canonical GT-AG splice sites, and their impact on pre-mRNA splicing was validated though functional assays. To our knowledge, this represents the first pathogenic case of a structural variant generating five distinct abnormal transcripts alongside the residual normal transcript, demonstrating a uniquely complex splicing pattern that differs from any previously reported variants. Ultimately, the elucidation of these complex molecular and splicing mechanisms highlights c.640-613ins1715 as a novel finding that significantly expands the mutational spectrum of the *GLA* gene.

**Table 3:** Pathogenic variant c.640-613ins1715 and other P/LP variants in intron4 of the *GLA* gene

No.	variants	Other names	location	Phenotype	Ref.
1	c.639+1 G > A	ND	ChrX:101400665	Classic	[15]
2	c.639+1 G > C	ND	ChrX:101400665	Classic	[16]
3	c.639+2T > C	ND	ChrX:101400664	Classic	[16]
4	c.639+4A > T	ND	ChrX:101400662	Classic	[17, 18]
5	c.639+5 G > A	ND	ChrX:101400661	Later-Onset	[18, 19]
6	c.639+5 G > T	ND	ChrX:101400661	Classic	[18, 20]
7	c.639+861C > T	c.640-859C > T	ChrX:101399805	Classic	[18, 21]
8	c.639+890T > C	c.640-830T > C	ChrX:101399776	ND	[18, 22]
9	c.639+919 G > A	c.640-801 G > A	ChrX:101399747	Later-Onset	[18, 23]
10	c.640-613ins1715	ND	ChrX:101399559	Classic	[9], this study
11	c.640-394C > T	IVS+1326C > T or c.639+1326C > T	ChrX:101399340	Later-Onset	[18, 24]
12	c.639+329_c.639+330ins113	ND	ChrX:101400337	Classic	[25]
13	c.640-11T > A	ND	ChrX:101398957	Classic	[18, 26]
14	c.640-3C > G	ND	ChrX:101398949	Classic	[18, 27]
15	c.640-2A > T	ND	ChrX:101398948	Classic	[18, 28]
16	c.640-2A > C	ND	ChrX:101398948	ND	[29]
17	c.640-2A > G	ND	ChrX:101398948	ND	[17]
18	c.640-1 G > A	ND	ChrX:101398947	Classic	[30]
19	c.640-1 G > C	ND	ChrX:101398947	Classic	[31]
20	c.640-1 G > T	ND	ChrX:101398947	Classic	[32]
21	c.640-1del	ND	ChrX:101398947	ND	[17]

### Splicing effect of deep intronic single nucleotide variants

The proband 2 carried two deep intronic single nucleotide variants (SNVs) in a cis configuration (cis c.640-363C > T, c.639+761 G > A) and presented with residual enzyme activity alongside a later-onset FD phenotype. In silico prediction tools (SpliceAI and the RDDC RNA Splicer) indicated that neither SNV would alter splicing (Table 1 and Figure S3). However, it should be emphasized that the computational algorithms are only predictive, and the true effect of these variants must be verified though functional studies. Therefore, three constructs were performed to validate the splicing impact of the individual variants (c.640-363C > T and c.639+761 G > A) as well as their cis configuration (cis c.640-363C > T, c.639+761 G > A), further elucidating the genotype-phenotype correlation.

**Fig. 3:**
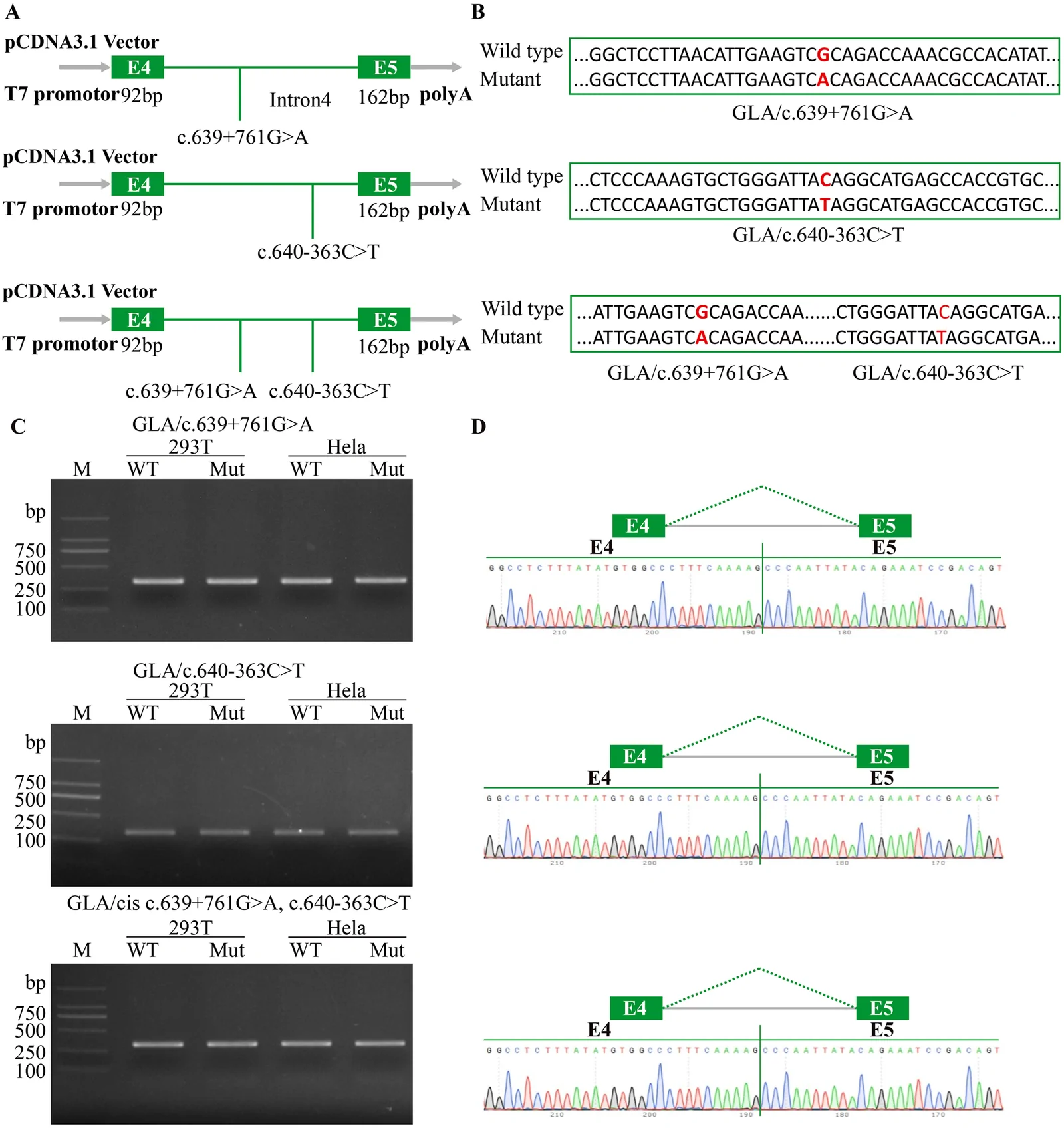
Minigene assay demonstrates the absence of splicing effects for variants c.639+761 G > A, c.640-363C > T, and c.639+761 G > A; c.640-363C > T in cis configuration in intron 4. (**A**) The schematic diagram of minigene constructs of the mutant. The green color shows fragments of the mutant genomic DNA and the gray color shows the pCDNA3.1 vector. (**B**) Genomic location of the variants. (**C**) RT-PCR products from 293 T and hela cells analyzed by agarose gel electrophoresis. (**D**) Schematic representation of splicing defects of variants and Sanger sequencing chromatograms of RT-PCR products

The minigene assays showed no aberrant splicing in any of the three constructs (Table 2, Fig. 3). These in vitro functional results demonstrated complete concordance with the in silico predictions. However, given the robust clinical diagnosis of a late-onset FD phenotype in this proband and the limitation of in vitro models, particularly the inherent risk of missing tissue-specific splicing events, both variants currently remain classified as VUS.

## Discussion

The diagnosis of FD remains challenging due to its highly variable clinical presentation and progressive multi-organ involvement. Genetic testing was used as the gold standard in clinical suspected FD patients, especially in females, whose residual enzyme activity frequently overlaps with that of healthy controls. While conventional Sanger sequencing and next-generation sequencing (NGS) are routinely employed for FD diagnosis, CAFD based on long-read sequencing exhibits a distinct advantage in resolving complex genetic landscapes, Nevertheless, in many patients, the underlying genetic etiology remains elusive. One of the reasons is that interpreting variants located within non-coding regions is still challenging. Although numerous in silico prediction tools are available, their reliability in evaluating deep-intronic variants, especially large structural variants like deletions and duplications remains fundamentally limited [34]. Consequently, determining the true pathogenic impact of putative splicing variants requires robust functional validation. While direct RNA sequencing (RNAseq) offers a straightforward and cost-effective approach to detect splicing and expression abnormalities, its clinical application is often limited. Specifically, mRNA extracted from peripheral leukocytes can sometimes be difficult to analyze owing to its low abundance and fragility. Thus, alternative functional assays (such as in vitro minigene models) are crucial for establishing the causality of complex non-coding variants.

In our study, systematic functional minigene assays were performed to elucidate the splicing effects of *GLA* variants identified via LRS in genetically unsolved patients. Although our prior in silico analyses predicted that the variant c.640-613ins1715 would disrupt RNA splicing, definitive functional evidence was previously absent. Our minigene assay successfully demonstrated that this insertion induces a complex array of aberrant splicing events, including partial exon skipping, intron retention, and PEs inclusion, and the activation of cryptic splice sites. The five resulting aberrant transcripts was predicted to introduce frame-shift and premature terminations codons (PTCs). If translated, these transcripts would yield truncated proteins lacking several functionally relevant domains; however, they are highly likely to undergo degradation via nonsense-mediated mRNA decay (NMD). Notably, the drastically reduced proportion of normal transcript corroborates the critically low functional protein levels, perfectly aligning with the proband’s classic FD phenotype.

To the best of our knowledge, this is the first report of a large, de novo, deep-intronic insertion generating five different aberrant transcripts associated with a classic FD genotype. Based on our in vitro functional validation, this variant was definitively classified as Pathogenic according to ACMG guidelines, fulfilling the PVS1 (Pathogenic very strong), PS2 (Pathogenic strong 2) and PS3 (Pathogenic strong 2) criteria. These findings underscore the indispensability of functional splicing validation in resolving variant pathogenicity and establishing accurate genotype-phenotype correlations to guide precision medicine.

As previously mentioned, approximately 5% *GLA* variants registered in HGMD are classified as splicing variants. With the improvement of detection technologies, this proportion is expected to rise. Notably, some deep-intronic variants, such as the prevalence Asian hotspot mutation c.936+919 G > A, highlight the clinical significance of these regions [35, 36]. Consequently, validating splicing effects is crucial for accurate pathogenicity interpretation and genotype-phenotype correlations. Among the 20 P/LP variants located in intron4, 50% (10/20) disrupt canonical splice sites (GT-AG). These variants are predicted to cause aberrant splicing because they impact highly conserved sequences that determine exon-intron boundaries [33, 34]. With the exception of three patients whose phenotypes were not documented, the remaining patients carrying these variants presented with the classic FD phenotype [15–17, 30–32].

The remaining 10 P/LP variants reside outside these canonical boundaries, necessitating functional assays to determine their potential impact on pre-mRNA splicing. To date, four such deep-intronic variants have been reported in intron 4 proximal to our identified variant (c.640-613ins1715): c.639+861C > T, c.639+890T > C, c.639+919 G > A and c.640-394C > T. Previous studies revealed that all four variants drive the inclusion of a 57bp cryptic PE, resulting in protein truncation alongside the production of residual normal transcripts [18, 24]. Notably, the three adjacent variants c.639+861C > T (Classic FD), c.639+890T > C (phenotype not documented), c.639+919 G > A (later-onset FD) consistently induce the same 57bp PE and resulting truncated protein (p.Lys213Pro214ins10*). The variant c.640-394C > T is more complex, generating two distinct aberrant transcripts encoding truncated proteins (p.Cys202* and p.Gly163Leufs*2) via a combination of cryptic PE inclusion and partial exon deletions. Although c.640-613ins1715 lies between c.639+919 G > A and c.640-394C > T, directly downstream of the 57-bp PE sequence, it does not generate transcripts containing this 57bp cryptic PE (Figure S2). Instead, this large insertion fundamentally reshape the local splicing landscape by altering existing canonical or cryptic splicing sites and introducing new ones, ultimately generating five distinct aberrant transcripts. Crucially, while all this deep intronic variants allow for the production normal transcripts, they result in marked clinical heterogeneity (ranging from classic to late-onset FD). The phenomenon highlights the complex contribution of varying aberrant transcript ratios to the pathogenesis of FD, reinforcing the critical necessity of functional validation for all non-canonical intronic variants.

Alu-Alu recombination is a well-documented mechanism contributing to large gene arrangements Among these rearrangements, 71.4% (5/7) were attributed to Alu-Alu recombination [3, 14, 30, 37, 38], while 14.3%(1/7) resulted from retrotransposition of an Alu element [3]. The remaining case was caused by Alu element-mediated insertion. The 5’ and 3’ breakpoints of these Alu-Alu recombination events were mapped to highly homologous Alu repeat elements (Figure S1), indicating that the four deletions [3, 30, 37, 38] and one duplication [14] were driven by homologous but unequal crossing over between Alu repeat elements, referred to Alu-Alu recombination. The Alu element-mediated insertion variant c.640-613ins1715 identified in the present study is a novel finding, which expands the mutational spectrum of the *GLA* gene and highlights the role of Alu elements in driving large insertions in deep intronic regions of *GLA* gene.

In contrast, the three constructs harboring c.640-363C > T, c.639+761 G > A and the cis configuration (c.640-363C > T, c.639+761 G > A) exhibited no significant effect on splicing in our in vitro minigene assay. However, assigning a benign classification to these variants remains premature. Given the proband’s later-onset FD phenotype, their slightly elevated but ambiguous allele frequencies In gnomAD (0.0004071 and 0.00008050, respectively), and the inherent risk of missing tissue-specific splicing events in an in vitro model, both variants remain classified as VUS. The discordance between the negative in vitro results and the patients’ disease status warrants careful consideration. It is possible that the pathogenic mechanism of these two SNVs in cis configuration are difficult to detect using a standard splicing assay due to the lack of genomic context. Furthermore, the splicing outcomes in patient-derived cells (and potentially across different cell types) may differ from our observations. Consequently, future studies employing diverse functional strategies, such as patient-derived fibroblasts and model organisms, are essential to elucidate the underlying pathological mechanisms. Nevertheless, while the lack of genomic text represents the limitation of minigene assays, previous studies have demonstrated the high reproducibility of splicing patterns generated by minigene constructs [18, 39]. Therefore, minigene assay remain a simple and high-capacity tool for functional analysis of putative splice variants.

Resolving the pathogenicity of such non-coding variants is of profound clinical significance. FD is now known to be more prevalent than initially thought, largely driven by the identification of late-onset cases [40–44], which have an estimated incidence of 1:4000 in recent newborn screening cohorts [45]. Definitive variant interpretation can confirm an FD diagnosis, enabling patients to access FD-specific treatment promptly. While symptomatic treatment remains is crucial for patients, it has no effect on the underlying pathophysiological progression of the disease. Ultimately, early initiation of FD-specific, disease-modifying treatments is critical to prevent irreversible organ damage and alter the natural history of FD [8].

## Conclusion

The results of this study underscore that functional assays are indispensable for accurately interpreting the pathogenicity of non-canonical variants. Such functional validation not only resolves ambiguities in variant classification but also enables precise molecular diagnosis in affected individuals, which are critical for genetic counseling and personalized therapeutic strategies.

## Electronic supplementary material

Below is the link to the electronic supplementary material.

Supplementary material 1

Supplementary material 2

## Data Availability

The datasets supporting the conclusions of this article are included within the article and its additional files.

## References

[1] Platt FM, d’Azzo A, Davidson BL, Neufeld EF, Tifft CJ. Lysosomal storage diseases. Nat Rev Dis Primers. 2018;4:27. 10.1038/s41572-018-0025-4. 30275469

[2] Germain DP. Fabry disease. Orphanet J Rare Dis. 2010;5(1):30. 10.1186/1750-1172-5-30. PMC3009617 21092187

[3] Dobrovolny R, Nazarenko I, Kim J, Doheny D, Desnick RJ. Detection of large gene rearrangements in X-linked genes by dosage analysis: identification of novel α-galactosidase a (GLA) deletions causing fabry disease. Hum Mutat. 2011;32(6):688–95. 10.1002/humu.21474. 21305660

[4] Kornreich R, Bishop DF, Desnick RJ. Alpha-galactosidase a gene rearrangements causing fabry disease. Identification of short direct repeats at breakpoints in an Alu-rich gene. J Biol Chem. 1990;265(16):9319–26. 10.1016/S0021-9258(19)38851-9. 2160973

[5] Zarate YA, Hopkin RJ. Fabry’s disease. Lancet. 2008;372(9647):1427–35. 10.1016/S0140-6736(08)61589-5. 18940466

[6] Arends M, Wanner C, Hughes D, Mehta A, Oder D, Watkinson OT, et al. Characterization of classical and nonclassical fabry disease: a multicenter study. J Am Soc Nephrol. 2017;28(5):1631–41. 10.1681/ASN.2016090964. PMC5407735 27979989

[7] Dobrovolny R, Dvorakova L, Ledvinova J, Magage S, Bultas J, Lubanda JC, et al. Relationship between X-inactivation and clinical involvement in fabry heterozygotes. Eleven novel mutations in the α-galactosidase a gene in the Czech and Slovak population. J Mol Med (Berl). 2005;83(8):647–54. 10.1007/s00109-005-0656-2. 15806320

[8] Palaiodimou L, Kokotis P, Zompola C, Papagiannopoulou G, Bakola E, Papadopoulou M, et al. Fabry disease: current and novel therapeutic strategies. A narrative review. Curr Neuropharmacol. 2023;21(3):440–56. 10.2174/1570159X20666220601124117. PMC10207921 35652398

[9] Yao F, Hao N, Li D, Zhang W, Zhou J, Qiu Z, et al. Long-read sequencing enables comprehensive molecular genetic diagnosis of fabry disease. Hum Genomics. 2024;18(1):133. 10.1186/s40246-024-00697-3. PMC11603755 39609713

[10] Richards S, Aziz N, Bale S, Bick D, Das S, Gastier-Foster J, et al. Standards and guidelines for the interpretation of sequence variants: a joint consensus recommendation of the American college of medical genetics and Genomics and the association for molecular pathology. Genet Med. 2015;17(5):405–24. 10.1038/gim.2015.30. PMC4544753 25741868

[11] Jaganathan K, Kyriazopoulou Panagiotopoulou S, McRae JF, Darbandi SF, Knowles D, Li YI, et al. Predicting splicing from primary sequence with deep learning. Cell. 2019;176(3):535–48.e24. 10.1016/j.cell.2018.12.015. 30661751

[12] Zhang Y, Bi S, Dai L, Zhao Y, Liu Y, Shi Z. Clinical report and genetic analysis of a Chinese neonate with craniofacial microsomia caused by a splicing variant of the splicing factor 3b subunit 2 gene. Mol Genet Evidence Med. 2023;11(12):e 2268. 10.1002/mgg3.2268. PMC10724505 37555391

[13] Banerjee S, Ren Y, Wei T, Zhou Z, Yu P, Guan F, et al. Next-generation sequencing detection and characterization of a heterozygous novel splice junction mutation in the 2B domain of KRT1 in a family with diffuse palmoplantar keratoderma. Exp Dermatol. 2015;24(2):152–55. 10.1111/exd.12610. 25429721

[14] Farr M, Ferreira S, Al-Dilaimi A, Bögeholz S, Goesmann A, Kalinowski J, et al. Fabry disease: detection of Alu-mediated exon duplication by NGS. Mol Cell Probes. 2019;45:79–83. 10.1016/j.mcp.2019.03.008. 30936019

[15] Rodríguez-Marí A, Coll MJ, Chabás A. Molecular analysis in fabry disease in Spain: fifteen novel GLA mutations and identification of a homozygous female. Hum Mutat. 2003;22(3):258. 10.1002/humu.9172. 12938095

[16] Ashton-Prolla P, Tong B, Shabbeer J, Astrin KH, Eng CM, Desnick RJ. Fabry disease: twenty-two novel mutations in the α-galactosidase a gene and Genotype/Phenotype correlations in severely and mildly affected hemizygotes and heterozygotes. J Investig Med. 2000;48(4):227–35. 10.1177/108155890004800403. 10916280

[17] Topaloglu AK, Ashley GA, Tong B, Shabbeer J, Astrin KH, Eng CM, et al. Twenty novel mutations in the α-galactosidase a gene causing Fabry disease. Mol Med. 1999;5(12):806–11. 10.1007/BF03401993. PMC2230489 10666480

[18] Okada E, Horinouchi T, Yamamura T, Aoto Y, Suzuki R, Ichikawa Y, et al. All reported non-canonical splice site variants in GLA cause aberrant splicing. Clin Exp Nephrol. 2023;27(9):737–46. 10.1007/s10157-023-02361-x. PMC10432374 37254000

[19] Choi JH, Lee BH, Heo SH, Kim GH, Kim YM, Kim DS, et al. Clinical characteristics and mutation spectrum of GLA in Korean patients with fabry disease by a nationwide survey: underdiagnosis of late-onset phenotype. Med (Baltim). 2017;96(29):e 7387. 10.1097/MD.0000000000007387. PMC5521888 28723748

[20] Zizzo C, Monte I, Pisani A, Fatuzzo P, Riccio E, Rodolico MS, et al. Molecular and clinical studies in five index cases with novel mutations in the GLA gene. Gene. 2016;578(1):100–04. 10.1016/j.gene.2015.12.024. 26691501

[21] Simoncini C, Torri S, Montano V, Chico L, Gruosso F, Tuttolomondo A, et al. Oxidative stress biomarkers in fabry disease: Is there a room for them? J Neurol. 2020;267(12):3741–52. 10.1007/s00415-020-10044-w. PMC7674365 32719972

[22] Lee SH, Li CF, Lin HY, Lin CH, Liu HC, Tsai SF, et al. High-throughput detection of common sequence variations of fabry disease in Taiwan using DNA mass spectrometry. Mol Genet Metab. 2014;111(4):507–12. 10.1016/j.ymgme.2014.02.004. 24613481

[23] Ishii S, Nakao S, Minamikawa-Tachino R, Desnick RJ, Fan JQ. Alternative splicing in the α-galactosidase a gene: increased exon inclusion results in the fabry cardiac phenotype. Am J Hum Genet. 2002;70(4):994–1002. 10.1086/339431. PMC379133 11828341

[24] Dai X, Zong X, Pan X, Lu W, Jiang GR, Lin F. Identification and functional characterization of the first deep intronic GLA mutation (IVS4+1326C>T) causing renal variant of fabry disease. Orphanet J Rare Dis. 2022;17(1):237. 10.1186/s13023-022-02377-8. PMC9208104 35725559

[25] Higuchi T, Kobayashi M, Ogata J, Kaneshiro E, Shimada Y, Kobayashi H, et al. Identification of cryptic novel α-galactosidase a gene mutations: abnormal mRNA splicing and large deletions. JIMD Rep. 2016;30:63–72. 10.1007/8904_2015_475 PMC5110446 27255140

[26] Lee BH, Heo SH, Kim GH, Park JY, Kim WS, Kang DH, et al. Mutations of the GLA gene in Korean patients with fabry disease and frequency of the E66Q allele as a functional variant in Korean newborns. J Hum Genet. 2010;55(8):512–17. 10.1038/jhg.2010.58. 20505683

[27] Nowak A, Mechtler TP, Hornemann T, Gawinecka J, Theswet E, Hilz MJ, et al. Genotype, phenotype and disease severity reflected by serum LysoGb3 levels in patients with fabry disease. Mol Genet Metab. 2018;123(2):148–53. 10.1016/j.ymgme.2017.07.002. 28728877

[28] Germain D, Biasotto M, Tosi M, Meo T, Kahn A, Poenaru L. Fluorescence-assisted mismatch analysis (FAMA) for exhaustive screening of the α-galactosidase a gene and detection of carriers in fabry disease. Hum Genet. 1996;98(6):719–26. 10.1007/s004390050292. 8931708

[29] Auray-Blais C, Blais CM, Ramaswami U, Boutin M, Germain DP, Dyack S, et al. Urinary biomarker investigation in children with fabry disease using tandem mass spectrometry. Clin Chim Acta. 2015;438:195–204. 10.1016/j.cca.2014.08.002. 25149322

[30] Shabbeer J, Yasuda M, Benson SD, Desnick RJ. Fabry disease: identification of 50 novel α-galactosidase a mutations causing the classic phenotype and three-dimensional structural analysis of 29 missense mutations. Hum Genomics. 2006;2(5):297–309. 10.1186/1479-7364-2-5-297. PMC3500179 16595074

[31] Cabrera G, Perretta F. Fabry disease. A potential pitfall a family with a novel intronic mutation. Mol Genet Metab Rep. 2018;17:16–17. 10.1016/j.ymgmr.2018.07.001. PMC6129647 30211005

[32] Sakuraba H, Tsukimura T, Togawa T, Tanaka T, Ohtsuka T, Sato A, et al. Fabry disease in a Japanese population-molecular and biochemical characteristics. Mol Genet Metab Rep. 2018;17:73–79. 10.1016/j.ymgmr.2018.10.004. PMC6205336 30386727

[33] Baralle D, Baralle M. Splicing in action: assessing disease causing sequence changes. J Med Genet. 2005;42(10):737–48. 10.1136/jmg.2004.029538. PMC1735933 16199547

[34] Anna A, Monika G. Splicing mutations in human genetic disorders: examples, detection, and confirmation. J Appl Genet. 2018;59(3):253–68. 10.1007/s13353-018-0444-7. PMC6060985 29680930

[35] Liu HC, Lin HY, Yang CF, Liao HC, Hsu TR, Lo CW, et al. Globotriaosylsphingosine (lyso-Gb3) might not be a reliable marker for monitoring the long-term therapeutic outcomes of enzyme replacement therapy for late-onset fabry patients with the Chinese hotspot mutation (IVS4+919G>A). Orphanet J Rare Dis. 2014;9(1):111. 10.1186/s13023-014-0111-y. PMC4223723 25047006

[36] Hwu WL. Deciphering the diagnostic dilemma: a comprehensive review of the Taiwanese cardiac variant in fabry disease. J Formos Med Assoc. 2024;123(7):738–43. 10.1016/j.jfma.2023.10.004. 37833114

[37] Ro LS, Chen CM, Chang HS, Lyu RK, Wu YR, Hsu WC, et al. Contribution of clinical screening to carrier detection in a large Chinese family with fabry disease due to a novel alpha-galactosidase a gene deletion. Eur J Neurol. 2007;14(5):493–97. 10.1111/j.1468-1331.2006.01611.x. 17437606

[38] Yoshimitsu M, Higuchi K, Miyata M, Devine S, Mattman A, Sirrs S, et al. Identification of novel mutations in the α-galactosidase a gene in patients with fabry disease: pitfalls of mutation analyses in patients with low α-galactosidase a activity. J Cardiol. 2011;57(3):345–53. 10.1016/j.jjcc.2010.12.004. 21333496

[39] Sanoguera-Miralles L, Valenzuela-Palomo A, Bueno-Martínez E, Esteban-Sánchez A, Lorca V, Llinares-Burguet I, et al. Systematic Minigene-based splicing analysis and tentative clinical classification of 52 CHEK2 splice-site variants. Clin Chem. 2024;70(1):319–38. 10.1093/clinchem/hvad125. 37725924

[40] Meikle PJ. Prevalence of lysosomal storage disorders. JAMA. 1999;281(3):249–54. 10.1001/jama.281.3.249. 9918480

[41] Morrone A, Cavicchi C, Bardelli T, Antuzzi D, Parini R, Di Rocco M, et al. Fabry disease: molecular studies in Italian patients and X inactivation analysis in manifesting carriers. J Med Genet. 2003;40(8):e103. 10.1136/jmg.40.8.e103. PMC1735554 12920095

[42] van der Tol L, Smid BE, Poorthuis BJ, Biegstraaten M, Deprez RH, Linthorst GE, et al. A systematic review on screening for fabry disease: prevalence of individuals with genetic variants of unknown significance. J Med Genet. 2014;51(1):1–9. 10.1136/jmedgenet-2013-101857. 23922385

[43] Chin SJ, Fuller M. Prevalence of lysosomal storage disorders in Australia from 2009 to 2020. Lancet Reg Health West Pac. 2022;19:100344. 10.1016/j.lanwpc.2021.100344. PMC8671750 35024668

[44] Gilchrist M, Casanova F, Tyrrell JS, Cannon S, Wood AR, Fife N, et al. Prevalence of fabry disease-causing variants in the UK biobank. J Med Genet. 2023;60(4):391–96. 10.1136/jmg-2022-108523. PMC10086508 35977816

[45] Spada M, Pagliardini S, Yasuda M, Tukel T, Thiagarajan G, Sakuraba H, et al. High incidence of later-onset fabry disease revealed by newborn screening. Am J Hum Genet. 2006;79(1):31–40. 10.1086/504601. PMC1474133 16773563

