# Supplementary material for: Improving molecular diagnosis of fabry disease: functional validation of novel splicing variants in GLA
Source: Orphanet J Rare Dis. 2026 Aug 13;21:413. doi: 10.1186/s13023-026-04512-1 (PMC13757196; doi:10.1186/s13023-026-04512-1)
Supplement: Supplementary file 1 [file 13023_2026_4512_MOESM1_ESM.docx]

**Figure S1 Structure of the *GLA* gene and the locations of variant c.640-613ins1715 and other reported large Alu-mediated intragenic rearrangements causing Fabry disease. Exons 1 through 7 are depicted as blue rectangles, and the positions of the 12 Alu repeat elements are indicated as gray rectangles. The locations and lengths of all known large Alu-mediated *GLA* intragenic rearrangements are shown. References for the reported gene rearrangements are provided below: c.194+1627_370-825del3445[1], c.195-1834_370-859del3151[2], c.207_369 + 651del814ins231[3], c.194+1562_369+1109del3443[4], c.195-1676_369+773del2619insCG[3], c.195-1193_640-560dup[5].**

**Figure S2 Genomic location of variant c.640-613ins1715 and other P/LP variants in intron4 of the *GLA* gene. Red and Green text indicates variants associated with classic and later-onset FD, respectively. Gray text represents variants with undocumented phenotypes. The blue box shows the 57-bp Pseudoexon (PE, ChrX:101,399,744-101,399,801.**

**Figure S3 The splicing predication in silico using the RDDC RNA splicer.**

**Reference：**

**1**.	Ro LS, Chen CM, Chang HS, Lyu RK, Wu YR, Hsu WC, et al. Contribution of clinical screening to carrier detection in a large Chinese family with Fabry disease due to a novel alpha-galactosidase A gene deletion. Eur J Neurol. 2007;14:493-497.

**2**.	Shabbeer J, Yasuda M, Benson SD, Desnick RJ. Fabry disease: identification of 50 novel alpha-galactosidase A mutations causing the classic phenotype and three-dimensional structural analysis of 29 missense mutations. Hum Genomics. 2006;2:297-309.

**3**.	Dobrovolny R, Nazarenko I, Kim J, Doheny D, Desnick RJ. Detection of large gene rearrangements in X-linked genes by dosage analysis: identification of novel α-galactosidase A (GLA) deletions causing Fabry disease. Hum Mutat. 2011;32:688-695.

**4**.	Yoshimitsu M, Higuchi K, Miyata M, Devine S, Mattman A, Sirrs S, et al. Identification of novel mutations in the α-galactosidase A gene in patients with Fabry disease: pitfalls of mutation analyses in patients with low α-galactosidase A activity. J Cardiol. 2011;57:345-353.

**5**.	Ferreira S, Valbuena C, Carvalho F, Oliveira JP. Novel human pathological mutations. Gene symbol: GLA. Disease: Fabry disease. Hum Genet. 2009;126:352.
